# Morphological function of toe fringe in the sand lizard *Phrynocephalus mystaceus*

**DOI:** 10.1038/s41598-020-79113-4

**Published:** 2020-12-16

**Authors:** Peng Zheng, Tao Liang, Jing An, Lei Shi

**Affiliations:** 1 College of Animal Science, Xinjiang Agricultural University, Urumqi, 830052 China; 2 Wildlife Conservation and Utilization, Nanjing Forestry University, Nanjing, 210037 Jiangsu China

**Keywords:** Zoology, Animal behaviour, Herpetology, Ecology, Behavioural ecology

## Abstract

Toe fringe is the most typical morphological feature of lizards adapted to sandy environments, and it is simple in shape, can evolve repeatedly, and has a high degree of repetition; therefore, this feature is suitable for testing the adaptive convergence suggested by form-environment correlations. *Phrynocephalus mystaceus* mainly lives in dune habitats, has a developed bilateral toe fringe, and exhibits fast sand-burying behavior for predator avoidance. We tested the effects of resecting the medial and bilateral toe fringes on the locomotor performance and sand-burying performance of *P. mystaceus.* The results showed that the maximum sprint speed and acceleration on sand substrate did not significantly differ under different conditions (*P* > 0.05). Sand-burying performance scores of the unresected individuals were significantly greater than those of the resected individuals (*P* < 0.05). A partial least squares (PLS) regression analysis showed that the relative area of toe fringe was the main factor affecting the sand-burying performance of unresected *P. mystaceus*. For lizards without fringe, the PLS regression showed that the swinging index of the hind-limb was the main factor affecting the sand-burying performance of the lizard. A comparison of the swinging indexes of the hind-limb of the lizard under three states revealed that under the unresected states, the frequency of the swinging of the hind-limb was significantly higher than those of lizards with resected bilateral fringes, further indicating that the lizards compensated for the loss of fringe by increasing the time and frequency of swinging of the hind-limb. A path analysis also showed that the fringe affected the sand-burying performance of *P. mystaceus* not only directly but also indirectly by affecting the frequency of the swinging of the hind-limb. After the bilateral toe fringe was removed, a significant negative correlation between locomotor and sand-burying performance was observed (*P* < 0.05). Taken together, these results provide experimental evidence that toe fringe is positively associated with the sand-burying performance of *P. mystaceus*.

## Introduction

Locomotion is a fundamental component of prey capture by and evasion of predators^1^, and an animal's escape behavior should reflect both the cost of interrupting current activities to respond to predators and the relative risk of predation^2,3^. Therefore, locomotion significantly affects the fitness of a species. Some lizards escape predators by rapidly burying themselves in sand^4–7^, and morphological differences can explain the diversity of behavior in many species^8^. According to a report about locomotor performance in *Anolis* lizard, individuals with larger hind limbs had better motor performance (jumping and sprinting)^9^. However, in a study of the climbing ability of *Anolis* lizard, individuals with shorter limbs had a better climbing performance^10^. Similar morphological characteristics for adaptation to the desert environment have evolved in different groups of desert lizards. One of the most common evolved morphological characteristics is toe fringe. According to Luke's research, “Lizard toe fringes are composed of laterally projecting elongated scales and have arisen independently at least 26 times in seven families of lizards”^11^. The results in studies of fringe and motion performance indicate that lizards with toe fringe run faster on sand surfaces than those without toe fringe; nevertheless, lizards with toe fringe exhibit slow speeds on rubber surfaces compared with individuals without fringe^12^. However, the role that fringe plays in the locomotor behavior of lizards is unclear. *Uma scoparia* have well-developed fringes that are thought to improve sprinting performance over fine sand^13,14^ . Studies have shown that the maximum running speed of lizards on sand significantly decreases after the fringe was removed. In particular, the locomotor performance of lizards was significantly decreased on uphill sloped surfaces^12^. Other studies have compared *Uma scoparia*, which has fringe, to *Callisaurus draconoides*, which does not have fringe, and found no significant differences in the sprint speeds on the different substrates^14–16^. In addition to locomotor performance, the fringe may also affect anti-predation behavior. For instance, *U. scoparia* uses its fringe for fast sand-burying behavior to escape predators and extreme heat^5,6^. *C. draconoides* and *U. scoparia* belong to different genera and show great differences in the relative limb proportions and potential behavioral, ecological, and physiological aspects. These variations complicate the examination of interspecific performance^12^. The above studies suggest that fringe may have a variety of functions. Therefore, whether a trade-off occurs between the various functions of fringe is also an issue of concern.

*Phrynocephalus mystaceus* is the largest species of *Phrynocephalus*, which is a genus of toad-headed agama lizards^17,18^. It is also a typical desert lizard species with a range from central Asia to northwest China, and it mainly lives in dune habitats and has a well-developed bilateral triangle toe fringe^11^. *P*. *mystaceus* can run quickly over fine sand substrates and exhibits fast sand-burying behavior when avoiding predators^5^. However, research on the morphology and locomotor performance of *P*. *mystaceus* is lacking. This species is listed as endangered on the Red List of China’s Vertebrates^19^. Therefore, studying the locomotor behavior of this species may help us better protect *P*. *mystaceus.* Although studies have removed the toe fringe of sand-dwelling lizards to test locomotor performance^13^, few studies have removed the toe fringe of sand-dwelling lizards to test their sand-burying performance. In this study, we measured several morphological traits and analyzed the locomotor and sand-burying performance of *P*. *mystaceus* on sand substrates. In particular, we adopted a control test that consisted of removing the toe fringe to verify the following scientific hypotheses: (1) The presence or absence of toe fringe on *P. mystaceus* will affect its locomotor performance over sand substrates. (2) The presence or absence of toe fringe on *P. mystaceus* will affect its sand-burying performance on sand substrates. (3) The toe fringe of *P. mystaceus* will influence the locomotor and sand-burying performance, and a trade-off occurs between the two performance levels.

## Materials and methods

In July 2018, we collected *P. mystaceus* individuals by hand from the Tukai Desert, Huocheng County, Yili Region, Xinjiang. Selected individuals in good condition were taken back to the Zoology Laboratory of Xinjiang Agricultural University. We measured the snout-vent length (SVL), head length (HL), head width (HW), head depth (HD), mouth breadth (MB), axilla-groin length (AG), abdominal width (AW), tail base width (TBW), fore limb length (FLL), hind-limb length (HLL), tail length (TL)^17^ using digital calipers. The mass was recorded on an electronic balance to the nearest 0.01 g, and the length of the forelimb and the hind-limb was divided into two parts: the humeral length (HL1) and radius length (RL) and femur length (FL) and tibia length (TL1). Because of the strong correlation between the fringe and toe length, we also measured all the toe lengths of the forelimb (FTL) and hind-limb (HTL) of *P*. *mystaceus*^20^.

All measurements were accurate to within 0.1 mm. The toe fringe of the lizards was quantified according to the following characteristic traits: individuals’ total fringe number divided by snout-vent length (TFN), individuals’ total fringe max length divided by snout-vent length (TFL), and individuals’ total fringe area divided by snout-vent length squared (TFA). We took pictures of the fringe characteristics with a Canon digital camera and measured and analyzed the data with image-pro Premier 6.0 software^21^. We housed animals individually in plastic terraria (30 × 20 × 20 cm, length × width × height). The plastic terraria were covered with 5 cm of fine sand collected from the original habitat of *P. mystaceus*, with a 60 W bulb suspended at one end as a heat source for thermoregulation. Enough *Tenebrio molitor* larvae and water supplemented with calcium and vitamins were provided to ensure that the animal received a full complement of nutrients. We allowed animals 1 week after arrival to acclimate to their new conditions before beginning the experimental trials. All tests were completed within 2 weeks.

**Figure 1:**
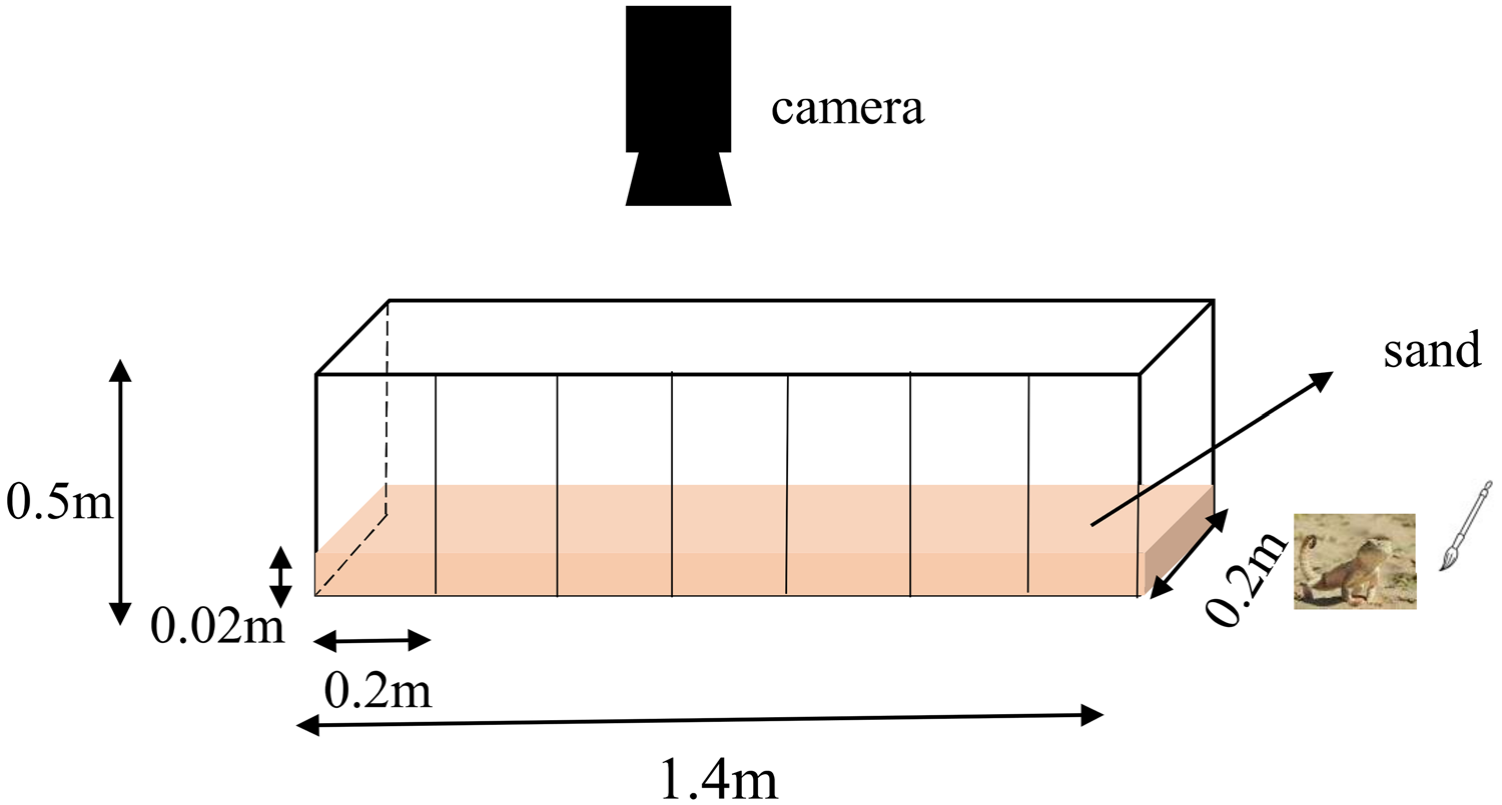
Diagrams of locomotor experimental materials, including the important dimensions (Lizard photo credit: Tao Liang).

The locomotor performance was measured on a 1.4 m horizontal track, and the racetrack was covered with 2 mm thick layer of sand substrate from the original habitat. Before testing locomotor performance, we conducted a preliminary test to determine the optimal temperature for the activity of *P. mystaceus*. We put ice water on one side of the track and adjusted the temperature in the track with a 60 W bulb in increments of 10 degrees to form a temperature gradient of 0°–50°. We moved the lizards onto the track, let the lizards adjust for 10 min, observed the active location of the lizard, and then used an infrared thermometer (Simzo HW-F7 China) to measure its temperature. Each individual was recorded three times, and the average value was the optimum activity temperature of *P. mystaceus*. We found that the optimal temperature for the activities of the great oared lizard was approximately 34 °C; thus, before the locomotor performance test, all individuals were preconditioned for 1 h at (34.0 ± 0.5) °C. Then, we moved the animal to the end of the track and used a brush to push it to sprint. A digital camera (Canon EOS 7d, FPS: 25) was used to record the lizard's movements on the track (Fig. 1). Through video playback, the 1.4 m track was divided into seven segments. In addition, the frames of each segment were counted, the motion time in the video was analyzed by Adobe premiere CS6 software, and then motion velocity of each segment was calculated based on the distance of each segment (0.2 m) divided by the motion time of each sections^22^. Finally, the maximum of seven values was used as the maximum sprint speed of *P. mystaceus.* The acceleration of each segment was calculated by Eq. (1). Finally, the maximum of seven values was used as the maximum acceleration of *P. mystaceus*.

1 $$ {\text{a}}_{{\text{c}}} = \left( {{\text{V}}_{{\text{c}}}^{2} - {\text{V}}_{{\text{s}}}^{2} } \right)/2{\text{S}} $$

where a_c_ = Current segment acceleration; V_c_ = Current segment motion velocity; V_s_ = previous segment motion velocity(if the current number of segments is 1, then this value is 0); and S = distance of current segment (0.2 m).

**Figure 2:**
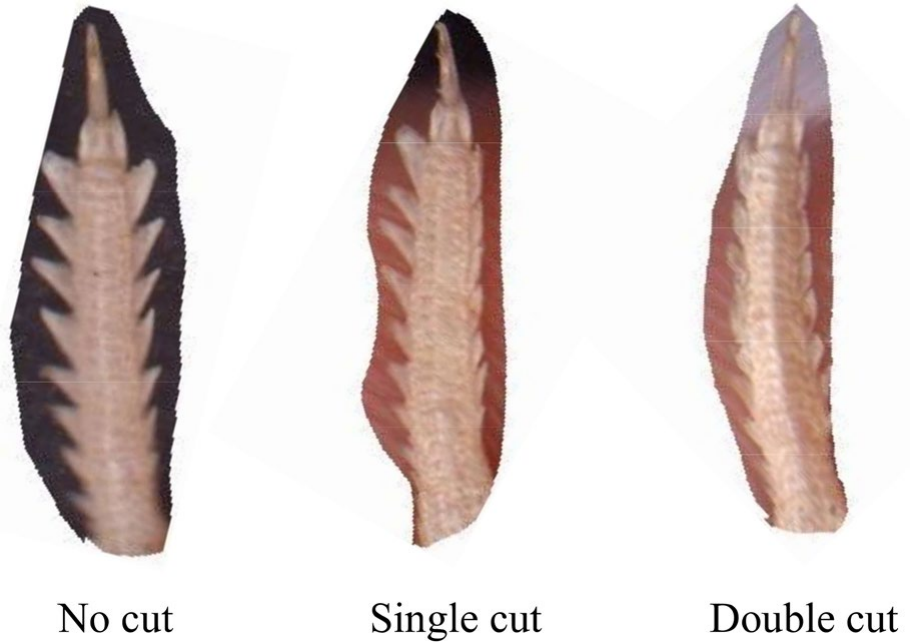
Pictures of fringe under three states (Photo credit: Peng Zheng).

The speed was graded by using an arithmetic sequence (to be divided by ten classes). The entire exercise test was divided into three repeats: uncut fringe, single cut (removal of the medial fringe), and double cut (removal of bilateral fringe) (Fig. 2). To avoid the effect of physical trauma caused by the removal the fringe on the movement and sand burying performance of the lizard, we paid close attention to the physical condition of the lizard when removing the fringes. If bleeding occurred, we stopped removing the fringe.

The sand-burying performance was measured in a tank (35 × 20 × 20 cm, length × width × height), and the bottom was covered with 10 cm of fine sand from the original habitat. We used a brush to stimulate the tail, which caused the individuals to dive into the sand. The entire process was recorded with a digital camera, and the sand-burying behavior was analyzed through video playback. The complete sand-burying behavior was also divided into three groups: uncut fringe, single cut and double cut. The sand-burying time was graded by an arithmetic sequence.

The scores of the sand-burying behavior were as follows: sand-burying ability score, sand-burying time score and comprehensive score. Among them, the sand-burying ability score was based on the sand-burying state of *P. mystaceus*: fully buried, 5 points; tail not buried, 4 points; head not buried, 3 points; most of the body not buried, 2 points; and not buried in the sand, 1 point. The sand-burying time score was used to sort the burying time from small to large according to the arithmetic sequence and divided into 5 grades, with the shortest burial time assigned a value of 5 points, and the points were successively divided into 4 points, 3 points, 2 points and 1 point according to the sorting method of the arithmetic sequence. The comprehensive score was the sum of the two score types. Individuals with the best and fastest burial performance had the highest overall score. In addition, the following sand-burying indicators were recorded: individual's total number of swings of the hind legs during the process of sand-burying (NHS, number of hind-limb swings), individual's total sand-burying time divided by sand-burying ability score (THS, time of hind-limb swings), individual's NHS divided by THS (FHS, frequency of hind-limb swing).

### Statistical analyses

The data were tested by Kolmogorov–Smirnov tests for detecting normality. The test results show that except for the FHS, all times were in accordance with the normal distribution. We log-transformed the variables to minimize the heterogeneity where necessary^23^. We performed a repeated measures ANOVA to examine the differences in locomotor performance, sand-burying performance and hind-limb swing index of the different states on the sand substrates with paired-sample t-tests (multiple comparisons). We corrected for multiple comparisons using the Benjamini–Hochberg method^24^. In terms of FHS, we performed an analysis of the nonparametric test to examine the differences in FHS of the different states on the sand substrates. Multiple regression is a suitable test for estimating the effects of morphological characteristics on the biological function of speed. However, the SVL, FLL, HLL, TL, TBW, MASS, TFN, TFL, and TFA tended to display high levels of multicollinearity, which can invalidate multiple regression analyses. Thus, the Farrar-Glauber test^25^ was used to assess whether extensive multicollinearity existed among these traits. Indeed, significant multicollinearity occurred for all nine log-transformed traits (Farrar Chi-Square = 88.456), Therefore, to assess the multiple effects of morphological traits on sprint speed while avoiding the issue of collinearity among the morphological traits, a partial least squares (PLS) regression^26^ was used to screen out the morphological characteristics affecting the performance of *P. mystaceus* during exercise and sand-burying. In the PLS regression, linear combinations of explanatory variables (morphology) are iteratively formed (PLS components), thereby maximizing the relationship to the response variable (speed)^27,28^. A path analysis can examine the simultaneous influence of several variables on a dependent variable and allows for greater specification of the relationships among variables^29^. It is a powerful method of analyzing the direct and indirect contributions of multiple comprehensive traits^30,31^. The use of path models is relatively common in ecology, evolution and organism biology^29,32^. Therefore, we conducted a path analysis implemented in Amos (v.24.0) to assess the morphological characteristics selected in the process of sand-burying to explore their internal relationships. Spearman’s correlation was used to analyze the correlation between the velocity score and the comprehensive score. All analyses except the path analysis were conducted using R v. 4.0.1^33^.

### Animal ethics

The following information pertains to the ethical approval (i.e., approving body and any reference numbers) for animal use: Specimens were collected and disposed of following the Guidelines for the Use of Live Amphibians and Reptiles in Field Research (the Herpetological Animal Care and Use Committee (HACC) of the American Society of Ichthyologists and Herpetologists, 2004). This study was conducted in compliance with current laws on animal welfare and research in China and the regulations set by Xinjiang Agricultural University. All experimental procedures involving animals were approved (animal protocol number: 2017012) by the Animal Welfare and Ethics Committee of Xinjiang Agricultural University, Urumqi, Xinjiang, China.

## Results

### Locomotor performance

**Figure 3:**
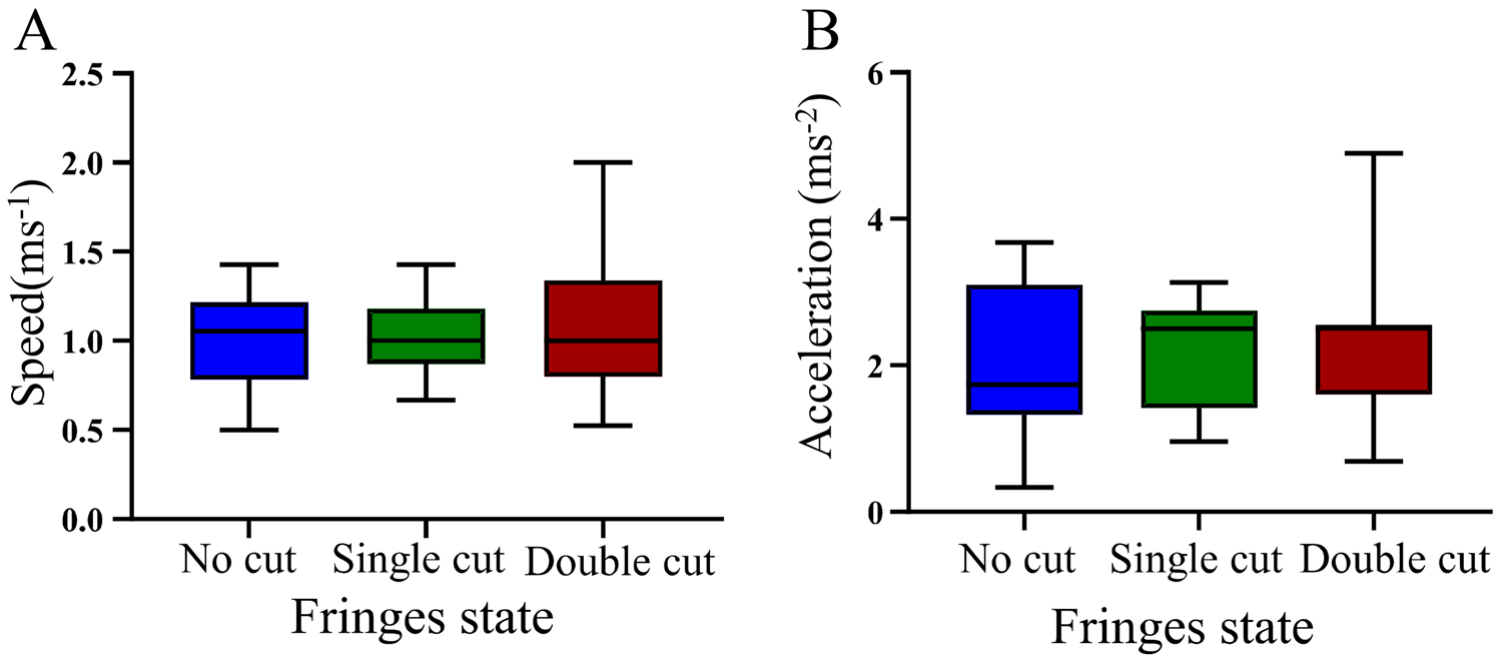
Maximum sprint speed (**A**) and maximum acceleration (**B**) over a sand substrate of *Phrynocephalus mystaceus* under different states. *Note* Different letters indicate significant differences at the *P* < 0.05 level.

In the running trials, significant differences were not observed in the maximum sprint speed on the sand substrate under the different conditions (repeated measures ANOVA, *F*_2, 24_ = 0.686, *P* = 0.520 Fig. 3A). The PLS regression analysis showed that under the uncut and double-cut states, due to the weak correlation between the maximum sprint speed and morphological characteristics, none of the morphological characteristics were the major determinants of locomotor performance. Under the single-cut state, SVL, HL, and MB were the major determinants of locomotor performance (Table 1). In terms of maximum acceleration, significant differences were not observed in the maximum acceleration on the sand substrate under the different conditions (repeated measures ANOVA, *F*_2, 24_ = 0.674, *P* = 0.525, Fig. 3B). The PLS regression analysis showed that under the double-cut states, no morphological characteristics were the major determinants of maximum acceleration. Under the uncut state, TL and HTL4 were the major determinants of maximum acceleration. Under the single-cut state, SVL, HL, HLL, TBW, MASS, and FL were the major determinants of maximum acceleration (Table 2).

**Table 1:** Effect of morphology on the maximum sprint speed under three states estimated using PLS.

State	Traits	Estimate	Std. error	t-value	Pr( >|t|)
Uncut	–	–	–	–	–
Single cut	SVL	0.108	0.029	3.694	0.006 **
	HL	0.067	0.027	2.499	0.036 *
	MB	0.053	0.021	2.488	0.037*
Double cut	–	–	–	–	–

Estimate represents the regression coefficients for the traits on speed. The standard error of the estimate (Std. Error), t values and probabilities (Pr( >|t|)) were estimated using the jack-knife procedure in the pls package of R. Significance levels: *P < 0.05, **P < 0.01.

**Table 2:** Effect of morphology on the maximum acceleration under three states estimated using PLS.

State	Traits	Estimate	Std. error	t-value	Pr( >|t|)
Uncut	TL	0.026	0.011	2.477	0.042*
	HTL4	0.025	0.009	2.516	0.040*
Single cut	SVL	0.102	0.039	2.586	0.032 *
	HL	0.048	0.021	2.361	0.045 *
	HLL	0.063	0.023	2.667	0.028*
	TBW	0.055	0.023	2.385	0.044*
	MASS	0.055	0.023	2.371	0.045*
	FL	0.093	0.040	2.329	0.048*
Double cut	–	–	–	–	–

Estimate represents the regression coefficients for the traits on speed. The standard error of the estimate (Std. error), t values and probabilities (Pr( >|t|)) were estimated using the jack knife procedure in the pls package of R. Significance levels: *P < 0.05, **P < 0.01.

### Sand-burying performance

**Figure 4:**
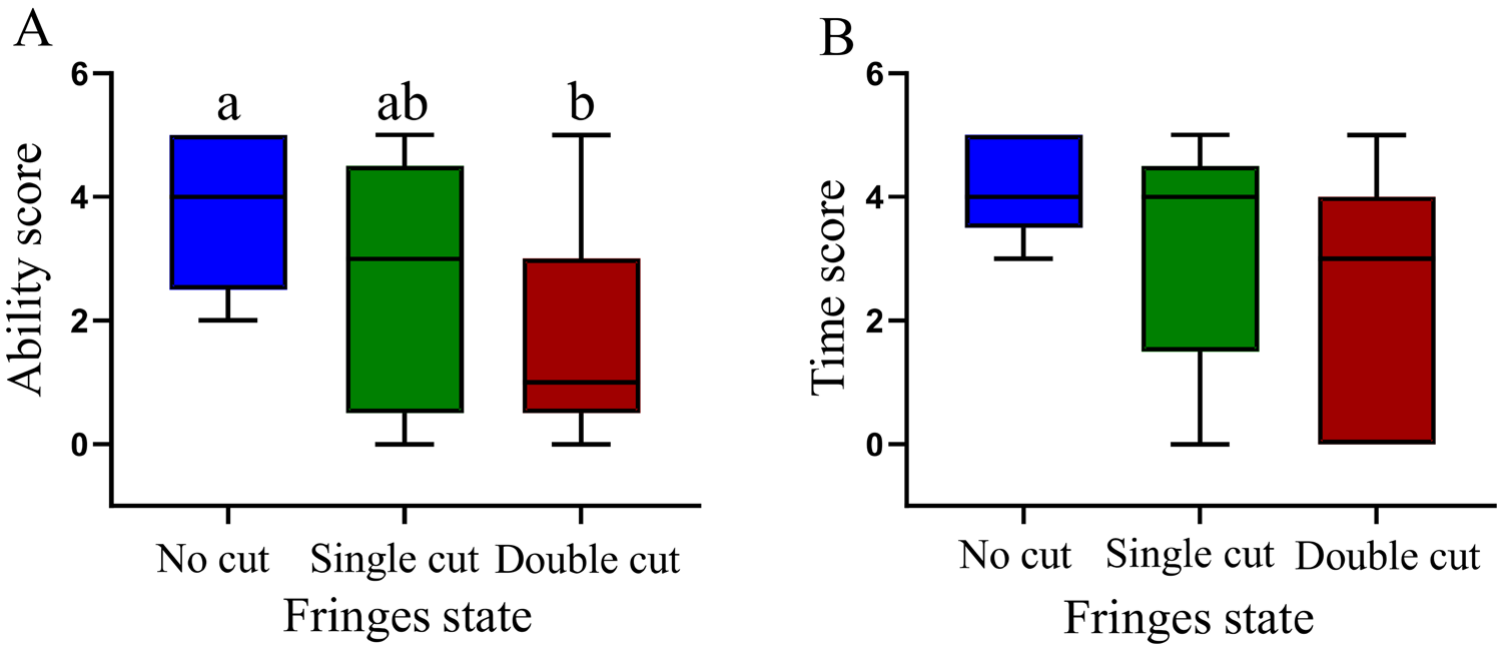
Ability scores (**A**) and time scores (**B**) for sand-burying by *Phrynocephalus mystaceus* under different states. Note: Different letters indicate significant differences at the *P* < 0.05 level.

The results of the univariate repeated measures ANOVA by sphericity test show that under different states, significant differences were observed in the sand-burying ability scores on the sand substrate (repeated measures ANOVA, *F*_2, 25_ = 3.718, *P* = 0.047), and the results of multiple comparisons (paired-sample t-test corrected by Benjamini–Hochberg method) showed that the sand-burying ability scores after removing the bilateral fringe were significantly lower than those before cutting (t = 3.333, *P* = 0.03, Fig. 4A). In addition, significant differences were not observed in the sand-burying ability scores under the other states (t = 2.06, *P* = 0.109; t = 0.77, *P* = 0.464, Fig. 4A). Significant differences were not observed in the sand-burying time scores on the sand substrate (repeated measures ANOVA, *F*_2, 25_ = 3.019, *P* = 0.077, Fig. 4B).

The results of PLS regression analysis showed that under the uncut state, TFA was the major determinant of locomotor performance. Under the single-cut state, HD, HLL, TL, FL, NHS, and THS were the key factors. Under the double-cut state, SVL, THS, and FHS were the major determinants of the sand-burying composite score (Table 3).

**Table 3:** Effect of morphology on the comprehensive score under the three states estimated using PLS.

State	Traits	Estimate	Std. error	t-value	Pr( >|t|)
No cut	TFA	0.181	0.064	2.803	0.023 *
Single cut	HD	0.048	0.012	4.119	0.006 **
	HLL	0.097	0.031	3.157	0.019 *
	TL	0.054	0.020	2.723	0.034 *
	FL	0.085	0.034	2.479	0.047*
	NHS	− 0.209	0.082	− 2.533	0.044 *
	THS	− 0.188	0.058	− 3.215	0.018*
Double cut	SVL	− 0.061	0.015	3.945	0.011 *
	THS	− 0.124	0.048	− 2.599	0.048 *
	FHS	0.098	0.023	4.256	0.008 **

Estimate represents the regression coefficients for the traits on the comprehensive score. The standard error of the estimate (Std. Error), t values and probabilities (Pr( >|t|)) were estimated using the jack knife procedure in the pls package of R. Significance levels: *P < 0.05, **P < 0.01.

**Figure 5:**
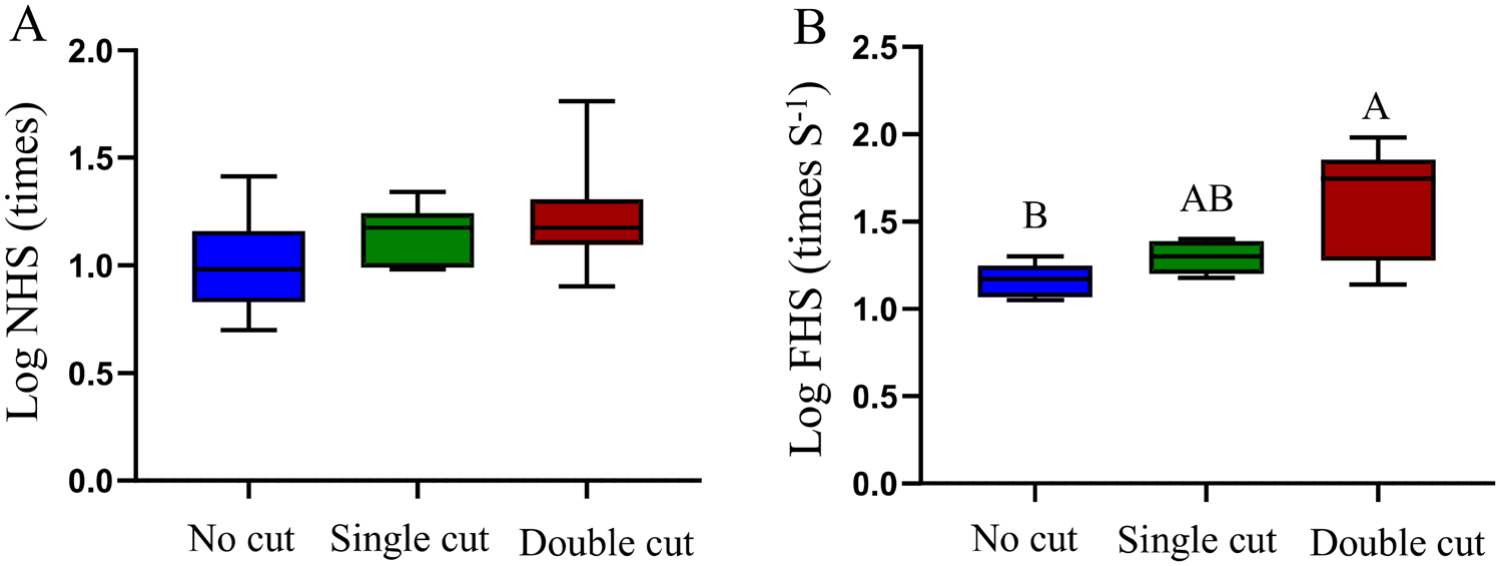
Log-transformed from the number of hind-limb swings (**A**) and log-transformed from frequency of hind-limb swing (**B**) of *Phrynocephalus mystaceus* with different fringe states. *Note* Different uppercase letters indicate extremely significant differences at the *P* < 0.01 level.

**Figure 6:**
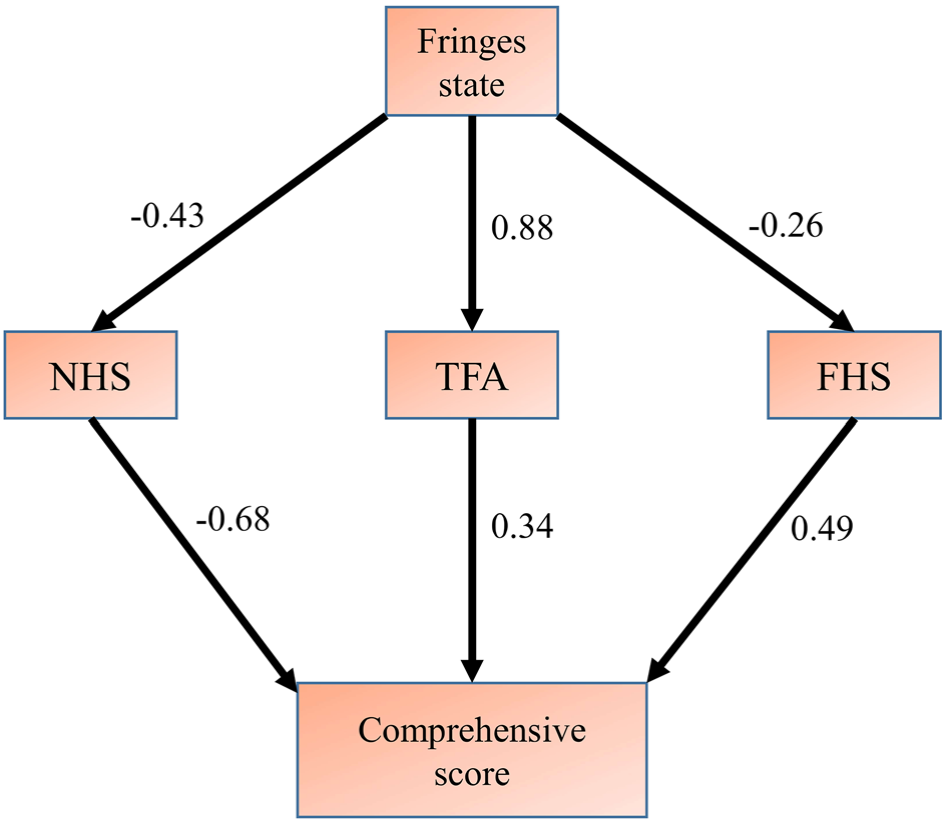
Path analysis diagram of sand-burying of *Phrynocephalus mystaceus*. Note: This path model examines how sand-burying is affected by the use of the fringe on the sand substrates. Numbers next to the path represent the relative contribution of the different fringe states during sand burying.

Under the different states, the most interesting finding is that significant differences were not observed in NHS on the sand substrate (repeated measures ANOVA, *F*_2, 18_ = 0.930, *P* = 0.443) (Fig. 5A). In terms of FHS, significant differences were observed among the three states (Nonparametric analysis, *X*^*2*^ = 6.076, *P* = 0.048). Further multiple comparison analyses (Benjamini–Hochberg method) showed that after removing the bilateral fringes when the lizard was buried in sand, the FHS was extremely significantly higher compared with the uncut state (adjusted *P* = 0.006, Fig. 5B). The results of the path analysis showed that differences in the state of the fringe not only directly affected the comprehensive score of sand-burying through TFA (Chi-square = 1.092, *P* < 0.01, Fig. 6, Table 4) but also had an effect on the NHS and FHS (Chi-square = 1.092, *P* < 0.01, Fig. 6, Table 4).

**Table 4:** Regression weights of the path analysis results about sand-burying of *Phrynocephalus mystaceus.*

Path label			Estimate	S.E	C.R	*P*
TFA	←	Fringe State	0.006	0.001	8.895	0.000***
NHS	←	Fringe State	− 0.115	0.052	− 2.219	0.026**
FHS	←	Fringe State	− 0.117	0.091	− 1.28	0.201
Comprehensive score	←	NHS	− 7.506	0.994	− 7.55	0.000***
Comprehensive score	←	TFA	141.741	38.528	3.679	0.000***
Comprehensive score	←	FHS	3.286	0.574	5.728	0.000***

*Note* Estimate represents the regression coefficients for each path. The standard error of the estimate (Std. Error), t value and probability (P) were estimated using the path analysis in Amos (v.24.0). Significance levels: *P < 0.05, **P < 0.01, ***P < 0.001.

**Figure 7:**
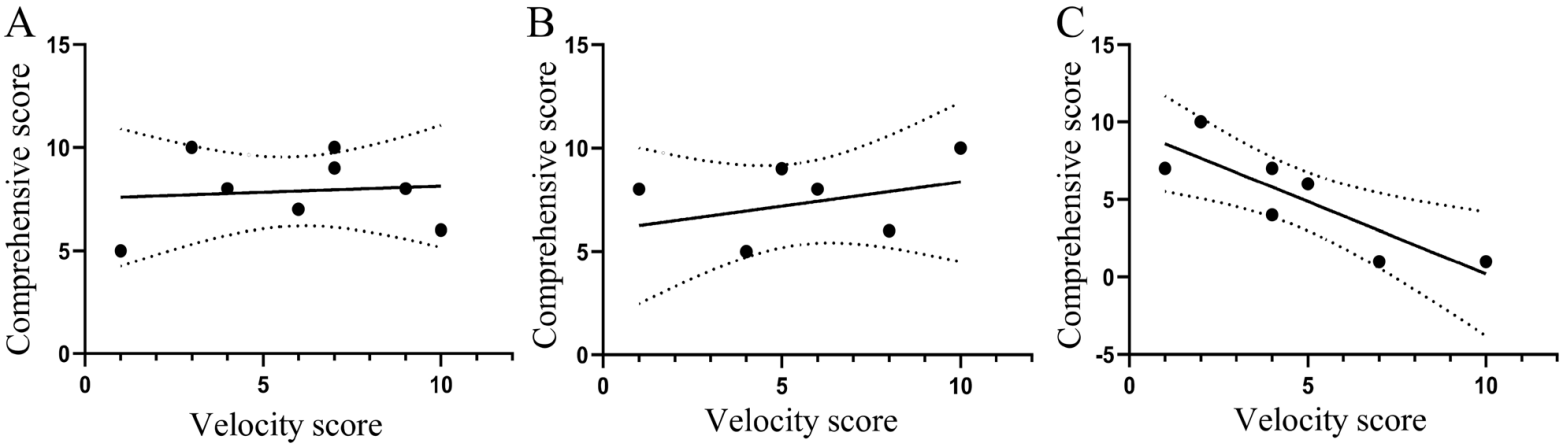
Correlation between the velocity score and comprehensive sand-burying score for *Phrynocephalus mystaceus* under the three fringe states. *Note* (**A**) uncut; (**B**): single cut; (**C**): double cut. The curve indicates the means with 95% confidence intervals.

In the case of no cutting, no significant correlations were observed between the velocity score and the comprehensive score (r^2^ = 0.03, *P* = 0.943, Fig. 7A). After removing the medial fringe, a significant negative correlation was not observed between the velocity score and the comprehensive score (r^2^ = 0.491, *P* = 0.263, Fig. 7B). However, in the case of double cutting, a significant negative correlation was observed between the velocity score and the comprehensive score (r^2^ = − 0.853, *P* = 0.015, Fig. 7C).

## Discussion

The morphological characteristics of animals are often related to their locomotor performance^34,35^. For example, the sprint speed of a lizard is related to body mass and tail size^20,36–39^. Hind-limb length and toe length are also thought to be indispensable factors for a lizard's sprint speed^40,41^. Natural selection acts on individual variation in locomotor performance in a given environment, thereby altering the trajectory of evolution in a variety of underlying traits governing locomotion^8,42–44^. In some cases, novel morphological structures have evolved that increase the performance of ecologically relevant tasks^45,46^. For example, *Rhoptropus afer* deploys adhesive toe pads to increase its speed on a level surface^47^. Our results reveal that for the single substrate (sand), significant differences were not observed in the locomotor performance of the lizard under the three states (Fig. 3A). This finding is similar to the results of “Carothers's experiment” in 1986, in which the locomotor performance of *Uma scoparia* was studied; Carothers found that the fringe did not affect the movement performance of lizard on rubber. None of the toe fringe indexes had any effect on the maximum sprint speed under the three fringes states. In terms of acceleration, the effect of fringe on the acceleration of the lizard is similar to that of the locomotor performance. Significant differences were not observed in the acceleration of the lizard under the three states (Fig. 3B). Compared with the special morphological feature of the fringes, the head shape and body size of *P. mystaceus* play a greater role in locomotor performance. For instance, male Asian house geckos (*Hemidactylus frenatus*) also experience a functional trade-off between bite force and sprint speed, with individuals with larger heads possessing greater bite strength but decreased speed from the heavier head^48^. These morphological characteristics may indicate that the locomotor performance of *P. mystaceus* is related to its bite force (that is, the trade-off between movement and bite force), which warrants further study.

Fringe is often thought to be an adaptation of lizards that allows for sand-burying. In *Uma* species, the fringe affects the sand movement as well as the sand-burying ability^49^. Sand-burying behavior is not only an antipredation behavior^50^ but also an adaptive behavior that is used to avoid environments that cause water loss and overheating^5,51^. The hind-limb is an important morphological feature that affects the locomotor performance of lizards^52,53^, and the fringe of the hind-limb of the *P. mystaceus* is more developed than that of other lizards. Our results show that under the condition in which sand-burying was likely to occur, the sand-burying performance when the fringe was not cut was significantly higher than when the bilateral fringe had been removed. However, the performance of *P. mystaceus* under the single cut condition was in the intermediate state. This finding further proves that the fringe significantly affected the sand-burying performance. In terms of fringe characteristics, under the uncut state, the relative TFA was selected by the PLS regression model of the sand-burying comprehensive score for *P. mystaceus*, and these scores were significantly positively correlated (Table 3). This result demonstrates that TFA promote the sand-burying performance of *P. mystaceus* and also confirms that the fringe of the lizard can act directly on the sand-burying behavior. Interestingly, after the medial fringe was removed, HD, HLL, TL, FL, NHS, and THS were the main characteristics affecting the sand-burying of the lizard. After the removal of the bilateral fringe, the sand-burying behavior of the lizard was only related to SVL, THS and FHS. We speculate that weakening the fringe function will indirectly increase the swing frequency of the hind-limb to compensate. To verify this conjecture, we compared the hind-limb swing indexes of *P. mystaceus* under the three states and found that FHS was significantly higher after bilateral fringe removal (Fig. 5B). Therefore, after the loss of fringe, *P. mystaceus* can complete sand-burying behavior by increasing the FHS, which further worsens the sand-burying time score. The longer FHS also increases the physical energy consumption by *P. mystaceus*. A possible explanation is that the fringe helps *P. mystaceus* bury in sand more easily. The path analysis results also showed that the fringe not only directly affects the performance of *P. mystaceus* but also indirectly affects the sand-burying performance by affecting the FHS (Fig. 6, Table 4).

Escape from predators by sand-burying is a common behavior for *P. mystaceus*. After the removal of the fringe, we found the lizards still tried to bury themselves completely in sand, For instance, although the lizards still have sand-burying behavior, the sand-burying ability score decreases obviously, and the increased THS and FHS suggest that more energy may be consumed. However, the well-developed toe fringe help *P. mystaceus* bury in sand quickly. The dry loose sand has similar properties to a fluid^54^, and adopting a swimming type of locomotion (e.g., undulatory) in this environment will reduce the energy expenditure during digging, which is typical adaptive behavior of sand lizards^55,56^. The effects of toe fringe on physiology, especially energy consumption and metabolic rate in the process of sand-burying, need to be confirmed by further study.

When single phenotypic traits perform multiple functions, phenomena such as functional redundancy, tradeoffs, and promotion are ubiquitous^57,58^. Sand-burying is a specific antipredation behavior that is related to the escape distance of *P. mystaceus*^50^. When confronted with danger in the wild, lizards that exhibit sand-burying behavior tend to prefer smooth surfaces and soft sand, which may be because this substrate requires less energy expenditure^59^. However, after the bilateral fringe was removed, a significant negative correlation was observed between running and sand-burying and the sand-burying ability score of the lizard decreased significantly. These changes will affect the lizard’s sand-burying efficiency and the choice of anti-predator strategy, with the lizard more likely to choose escape than burial. These findings indicate that the toe fringe of *P. mystaceus* is important for sand-burying behavior.

Male *P. mystaceus* individuals spend considerable time looking around and sunbathing at the top of sand dunes (personal observation), and they defend the field by exhibiting threat behavior and fighting to chase away intruders. In the face of threats from natural enemies, *P. mystaceus* can choose to run or bury themselves in the sand to avoid the enemy. Studies have shown that sand-burying behavior can reduce the risk associated with the occupation of exposed areas^6,60^. Therefore, the effect of fringe on the sand-burying performance of *P. mystaceus* has adaptive value. The repeated evolution of fringe among different groups of lizards and their relationship to specific environments^11,13,14,50^ strongly support adaptive explanations involving movement (and burying) in sandy environments. In addition, toe fringe has been derived in the *Uma* genus^61^, which is probably due to adaptations to sand-bearing environments^12^. Molecular phylogenetic studies based on mitochondrial gene fragments have shown that *P. mystaceus* belongs to the primitive group in *Phrynocephalus*^62,63^. Except for *P. mystaceus*, Other *Phrynocephalus* species (with less developed fringe) show sand-burying behaviors but have difficulty completing the task^5^. However, recent studies based on nuclear genes (nuDNA) suggest that the location of the base position of *P. mystaceus* is the result of interspecific hybridization and ancient mitochondrial gene infiltration. The common ancestor of *Phrynocephalus* probably preferred sandy substrates with the inclusion of clay or gravel. Climate change in the middle Miocene led to the migration of *Phrynocephalus* lizards into the desert and the diffusion and adaptive evolution of large wind-blown dune habitats^18^. If this is the case, then the fringe of the *Phrynocephalus*, which is similar to that of *Uma*, is also the result of convergent evolution to adapt to the sand environment.

## Conclusions

The toe fringe of *P. mystaceus* plays a significant role in its antipredation behavior but have little effect on its locomotor performance. However, the species exhibits better sand-burying performance under the bilateral fringe state. Moreover, TFA can promote the burying performance of *P. mystaceus*. The fringe also plays an important role in the selection of antipredation strategies. In the three fringe states, the predator avoidance strategy of *P. mystaceus* gradually shifted from sand-burying to locomotion. These findings show that the fringe plays a considerably role in sand-burying behavior.
